# Belgian psychiatrists’ attitudes towards, and readiness to engage in, euthanasia assessment procedures with adults with psychiatric conditions: a survey

**DOI:** 10.1186/s12888-020-02775-x

**Published:** 2020-07-16

**Authors:** Monica Verhofstadt, Kurt Audenaert, Kris Van den Broeck, Luc Deliens, Freddy Mortier, Koen Titeca, Koen Pardon, Kenneth Chambaere

**Affiliations:** 1 End-of-Life Care Research Group, Vrije Universiteit Brussel (VUB), Brussel, Belgium; 2 End-of-Life Care Research Group, Department Public Health and Primary Care, Ghent University, Corneel Heymanslaan 10, 6K3, Ghent, 1000 Belgium; 3 Department of Psychiatry, Ghent University Hospital, Ghent, Belgium; 4 Collaborative Antwerp Psychiatric Research Institute (CAPRI), Antwerp University, Antwerp, Belgium; 5 Bioethics Institute Ghent, Ghent University, Ghent, Belgium; 6 Department of Psychiatry, General Hospital Groeninge, Courtrai, Belgium; 7 ULteam, end-of-life consultation centre, Wemmel, Brussels, Belgium

**Keywords:** Euthanasia, Mental disorders, Assisted suicide, Psychiatry, Survey study

## Abstract

**Background:**

Although the Belgian assessment pathway for legal euthanasia requires the engagement of at least one psychiatrist, little is known about psychiatrists’ attitudes towards euthanasia for adults with psychiatric conditions (APC). This study aims to gauge psychiatrists’ attitudes towards and readiness to engage in euthanasia assessment and/or performance procedures in APC.

**Methods:**

This cross-sectional survey study was performed between November 2018 and April 2019. The survey was sent to a sample of 499 eligible psychiatrists affiliated to the Flemish Association for Psychiatry, a professional association that aims to unite and represent all psychiatrists working in Flanders, the Dutch-speaking, northern part of Belgium. The Association’s members comprise an estimated 80–90% of all psychiatrists active in Flanders. Only psychiatrists working with APC (83% of the association’s total membership) were included. Factorial Anova and Chi Square tests were performed to examine if and to what extent psychiatrists’ backgrounds were associated with, respectively, their attitudes and their readiness to play a role in euthanasia procedures concerning APC.

**Results:**

One hundred eighty-four psychiatrists completed the questionnaire (response rate 40.2%); 74.5% agree that euthanasia should remain permissible for APC. However, 68.9% question some of the approaches taken by other physicians during the euthanasia assessment and only half consider euthanasia assessment procedures compatible with the psychiatric care relationship. Where active engagement is concerned, an informal referral (68%) or preliminary advisory role (43.8%) is preferred to a formal role as a legally required advising physician (30.3%), let alone as performing physician (< 10%).

**Conclusion:**

Although three quarters agree with maintaining the legal option of euthanasia for APC, their readiness to take a formal role in euthanasia procedures appears to be limited. More insight is required into the barriers preventing engagement and what psychiatrists need, be it education or clarification of the legal requirements, to ensure that patients can have their euthanasia requests assessed adequately.

## Background

Since 2002, Belgium has provided a legal framework which – under certain conditions – enables patients suffering illnesses, including psychiatric disorders, to choose to die by means of euthanasia. No former Belgian research endeavour has focused on the attitudes of psychiatrists towards euthanasia for adults with psychiatric conditions (APC). This is striking, as the Belgian legal procedure for euthanasia assessment requires the consultation of at least one psychiatrist for this specific patient group and research outside of Belgium has revealed strong reservations among psychiatrists toward euthanasia in APC.

Euthanasia (the administering by a physician of life-ending drugs to the patient at the latter’s request) and/or physician-assisted suicide (where a physician prescribes and provides life-ending drugs to the patient, at their own request, for the patient to self-administer) is legal in a small number of countries worldwide and some US states, and mainly applies to those who are terminally ill [1–3]. There are only a few European countries (Belgium, Luxembourg, Switzerland and the Netherlands) where euthanasia requests from non-terminally-ill patients can be granted when based primarily on psychiatric conditions [1]. In Belgium, the act of euthanasia is only legal when all the legal criteria are fulfilled (see Box 1 in OSF). The Belgian law does not explicitly cover physician-assisted suicide. However, in its information brochure for physicians, the Federal Control and Evaluation Commission for Euthanasia has stated that, in their opinion, physician-assisted suicide is also covered by the Euthanasia Law as the Law does not prescribe how euthanasia should be performed [4]. In any case, physician-assisted suicide is extremely rare in Belgium.

Since the Belgian Law on Euthanasia came into effect in 2002, reported euthanasia rates based on psychiatric conditions (other than dementia) have risen from five cases in the first five years of the euthanasia law and 72 cases in the second to 181 cases in the third [5, 6]. The numbers represent a proportional increase from 0. 25% of all reported euthanasia cases in APC between 2002 and 2005 to 2.1% in 2015, with a decrease to 1.2% between 2016 and 2017.

The increasing number of euthanasia cases based on psychiatric conditions has generated increasing ethical and societal debate. Public media have reported on controversial euthanasia cases based on psychiatric conditions – three of which were referred to Belgian or European Courts [7–9] – and some observers have expressed concerns about potentially overly-permissive approaches in some instances, in terms of the assessment of eligibility [10]. Therefore, health care institutions and ethical and professional organisations have recently developed and published guidelines on the assessment of euthanasia requests in APC emphasizing the need for careful scrutiny in euthanasia decision-making procedures [11].

Psychiatrists are key players in euthanasia as regards APC [12–14], as the Law on Euthanasia clearly states that they should be involved, at least as advising physicians, when patients request euthanasia in cases of non-terminal (neuro) psychiatric disease. However, to date, little is known about how they feel about euthanasia in APC, and to what extent they are prepared to be involved in such procedures.

Currently, only the Netherlands provides information from periodic evaluation and survey studies on euthanasia practice involving APC from a psychiatrist’s perspective and results show that although the number of euthanasia cases performed has increased over time, the Dutch professional body of psychiatrists has become more reluctant to engage in or grant euthanasia requests from APC over the years [15, 16]. Recent cross-sectional studies gauging Canadian and Swiss psychiatrists’ attitudes to such cases also show this reluctance [17, 18]. This is commonly attributed to the complexity and difficulty of adequately assessing all legal substantive criteria in APC [15, 19].

This study aims to complement both the little knowledge that exists and the current debate with findings from Belgian euthanasia practice. We will address the following research questions:

1. What are Dutch-speaking psychiatrists’ attitudes towards euthanasia and the practice of euthanasia in APC? To what extent are their attitudes related to their personal and professional characteristics?
2. To what extent would Dutch-speaking psychiatrists consider being involved in the assessment and/or performance of euthanasia procedures regarding APC? And to what extent is their willingness/unwillingness to be involved in such euthanasia procedures related to their personal and professional characteristics?

## Methods

### Study design

This cross-sectional study consisted of a paper and web survey on psychiatrists’ attitudes towards and readiness to be involved in euthanasia requests and procedures for APC.

### Participants

As Belgium is divided into Flanders (the Dutch-speaking region in the north), Wallonia (the French-speaking region in the south), and Brussels (the capital, which is officially bilingual), the survey was launched among the Dutch-speaking psychiatrists. According to the latest report of the Federal Control and Evaluation Commission for Euthanasia, the ratio of performed euthanasia cases in the French-Speaking versus the Dutch-speaking region has been 20/80 [6].

The total sample consisted of 600 psychiatrists, all members of the Flemish Association for Psychiatry (Vlaamse Vereniging voor Psychiatrie, VVP), a professional body that aims to unite and represent all psychiatrists working in Flanders, the Dutch-speaking northern part of Belgium. VVP-affiliated psychiatrists made up approximately 47% of the total professional group of registered psychiatrists in Flanders (1286, of whom 910 were registered in the database of the National Institute for Sickness and Disability Insurance [20]. The distribution of the survey was limited to Dutch-speaking psychiatrists affiliated to the VVP for practical reasons (see OSF).

### Survey instrument

For this study, survey questions on psychiatrists’ attitudes and readiness to be engaged in euthanasia procedures in APC were taken from a larger survey instrument that is posted in the Open Science Framework repository (see appendix A and B in OSF) accompanying this paper (in Dutch). The larger survey not only aimed to examine psychiatrists’ attitudes, but also their concrete experiences and whether they can see themselves taking part in euthanasia procedures based on psychiatric conditions in the future. The instrument was developed on the basis of five existing questionnaires [15, 16, 21–23], and adjusted to the context of current psychiatric clinical practice in Flanders.

The draft survey was presented at a meeting of 15 psychiatrists from the psychiatry ward of Ghent University Hospital for cognitive validation purposes (i.e. for participants to identify potential problems as regards item interpretation, item redundancy, *completeness of the survey*, feasibility to generate correct answers, and time estimation). Finally, the survey was revised by the members of the broader research group (for more details, see the research protocol in OSF idem, appendix C).

For this specific study, the following 12 items of the larger survey (see OSF idem, appendix D) were selected and divided into three main parts: 1) seven items covering the psychiatrist’s personal and professional background; 2) one item consisting of 13 statements on attitudes towards euthanasia, to rate on a 5-point Likert scale, ranging from ‘totally disagree’ to ‘totally agree’, e.g. ‘euthanasia should be legal for psychiatric patients’; 3) three items on psychiatrists’ readiness to take up one or more roles in euthanasia procedures concerning APC (as e.g. the treating, advising and/or performing physician in the future; see Box 2 for a helpful glossary, explaining each of the active roles a psychiatrist could be engaged in); and 4) one open-ended question at the end of the questionnaire, consisting of a comment box, in which responding psychiatrists could clarify their answers if necessary.

### Ethics

This research project was performed in accordance with the Declaration of Helsinki and received ethical approval from the Medical Ethics Committee of the Brussels University Hospital with reference BUN 143201837302 and the Medical Ethics Committee of the Ghent University Hospital with reference 2018–1165.

### Procedure

#### Data collection

*The VVP members* were invited by e-mail to participate in the study. A link to LimeSurvey’s online platform [24] was included and the information letter (see OSF idem, appendices E and F, in Dutch) attached. Data were collected between November 2018 and April 2019. Non-responders received a first reminder by e-mail, two weeks after the initial invitation. A second reminder, including a paper-and-pencil version of the questionnaire, was sent by post three weeks after the survey was launched.

### Analyses and criteria

Data were imported from LimeSurvey into SPSS version 25 and cleaned according to the principles of a data analysis plan (OSF idem, Appendix G). The SPSS database was supplemented with data gathered from the returned paper surveys. Missing data were excluded from analyses.

To describe the sample, we calculated aggregated descriptives on the psychiatrists’ personal and professional characteristics that were also used as independent variables in further statistical analyses. Factorial Anova and Chi Square tests were performed to examine if and to what extent psychiatrists’ backgrounds were associated with, respectively, their attitudes and their readiness to take a role in euthanasia procedures concerning APC. See appendix G in OSF for the syntax used for hypothesis testing. The open question at the end was checked for relevant answers, thematically analysed and included in the findings.

## Results

### Description of the sample

The VVP consisted of 600 members; 101 members were not professionally active as psychiatrists and/or had no work experience with adult psychiatric patients. The response sample consisted of 201 out of 499 psychiatrists (valid response rate 40.2%). The data from 17 psychiatrists were excluded from further analysis, for example due to no explicit agreement regarding informed consent or too many missing answers. The responses of 184 psychiatrists were found eligible for further analysis, including data from retired psychiatrists as they can still be involved in euthanasia procedures.

Table 1 shows the characteristics of the responding psychiatrists. Of all psychiatrists, 56.5% were male. The majority (66.8%) worked in a psychiatric hospital care facility, 45.7% in a private practice and 13% in a community mental healthcare centre; 17.9% had less than five years of work experience – they were trainees in psychiatry – and 48.4% had more than 20 years of work experience. Nine (4.9%) had received special training in end of life matters, while 91 (49.5%) felt competent to be involved in euthanasia procedures.

**Table 1:** Psychiatrists’ demographics and professional characteristics

Gender	Sample (*N* = 184)	
	n	%
Male	104	56.5
Female	77	41.8
Unknown	3	1.6
Age (in years)	n	%
< 30	28	15.2
30–40 years	40	21.7
41–60 years	65	35.3
> 60	51	27.7
Worked as psychiatrist or psychiatric trainee during last year	n	%
Yes	167	90.8
No	16	8.7
Missing	1	0.5
Clinical setting^a^	n	%
Private or Group Practice	84	45.7
Psychiatric Hospital Care	123	66.8
Community Mental HealthCare Center	24	13.0
Psychiatric Nursing Home	9	4.9
Psychiatric Home Care	6	3.3
Sheltered housing	13	7.1
Other^b^	26	14.1
Work experience (in number of years)	n	%
< 5 years	33	17.9
6–10 years	20	10.9
11–20 years	42	22.8
> 20 years	89	48.4
Ever received special training in End Of Life care	n	%
Yes	9	4.9
No	173	94.0
Missing	2	1.1
Feels competent to be involved in euthanasia procedure		
Yes	91	49.5
No	92	50
Missing	1	0.5

^a^ Some psychiatrists had more than one workplace

^b^ Other work places: prison or forensic psychiatric centers, psychiatric and psychosocial rehabilitation centers, psychiatric mobile crisis or response teams, other housing and care centers for other subpopulations (e.g. students, disabled persons)

### Attitudes to euthanasia

As illustrated in detail in Table 2, a minority of psychiatrists (29.9%) were in favour of restricting euthanasia, as a legal end-of-life option, to the terminally ill. According to the majority of psychiatrists (74.5%), euthanasia should remain legal for APC.

**Table 2:** Psychiatrists’ attitudes toward euthanasia in general and in psychiatry

Attitude statements	Response in N/%^a^					Combined percentages
	Totally disagree	Disagree	Neutral	Agree	Totally agree	Agree + Totally agree
St1: Euthanasia should only be legally allowed for the terminally ill.	40 21.7%	70 38.0%	19 10.3%	32 17.4%	23 12.5%	55 29.9%
St2: Euthanasia should be legally allowed for the non-terminally ill, but only when based on somatic illnesses.	53 28.8%	91 49.5%	20 10.9%	13 7.1%	7 3.8%	20 10.9%
St3: Euthanasia should remain legally allowed for patients with psychiatric illnesses.	17 9.2%	19 10.3%	11 6.0%	68 37.0%	69 37.5%	137 74.5%
St4: A psychiatric patient can suffer unbearably.	2 1.1%	1 0.5%	7 3.8%	37 20.1%	137 74.5%	174 94.6%
St5: A psychiatric patient’s death request can be well considered, and not only considered as a symptom of the patient’s psychopathology.	4 2.2%	8 4.3%	10 5.4%	92 50.0%	70 38.0%	162 88%
St6: A psychiatric patient can find herself in a medically hopeless situation.	5 2.7%	9 4.9%	16 8.7%	67 36.4%	87 47.3%	154 83.7%
St7: For a psychiatric patient, a lack of reasonable treatment perspectives can exist.	2 1.1%	23 12.5%	17 9.2%	76 41.3%	66 35.9%	142 77.2%
St8: Euthanasia assessment in psychiatric patients is compatible with a psychotherapeutic relationship.	26 14.1%	31 16.8%	30 16.3%	60 32.6%	37 20.1%	97 52.7%
St9: During the assessment of a psychiatric patient’s euthanasia request, potentially effective therapeutic treatment options should be taken into account.	7 3.8%	34 18.5%	36 19.6%	74 40.2%	33 17.9%	107 58.1%
St10: During the assessment of a psychiatric patient’s euthanasia request, the focus should not only be placed on the patient’s medical condition, but also on the patient’s whole life context.	6 3.3%	10 5.4%	19 10.3%	77 41.8%	72 39.1%	149 80.9%
St11: Euthanasia is an acceptable alternative to prevent for suicide.^b^	35 19.1%	33 18.0%	35 19.1%	64 35.0%	16 8.7%	80 43.7%
St12: In psychiatric patients, physician-assisted suicide (physician provides the lethal drugs to the patient who then self-administers it) is more acceptable than euthanasia (physician administers the lethal drugs to the patient).	36 19.6%	41 22.3%	49 26.6%	46 25.0%	12 6.5%	58 31.5%
St13: In some cases, there is mention of psychiatric euthanasia assessment that was too lightly dealth with.^c^	2 1.1%	16 8.7%	39 21.3%	66 36.1%	60 32.8%	126 68.9%

^a^ Range Likert scale: from 1 “totally disagree” to 5 “totally agree”. For all items: Minimum score = 1 and maximum score = 5

^b^ Missings: *n* = 2 (St11 and St13: *n* = 1, missings from 2 different psychiatrists)

As regards the eligibility of a psychiatric patient’s euthanasia request, the majority agreed that APCs can suffer unbearably (94.6%), can make a well-considered euthanasia request (88%), and can find themselves in a medically hopeless situation (83.7%) as a lack of reasonable treatment perspectives can exist (77.2%).

With regard to the assessment of an APC’s euthanasia request, about half of the psychiatrists (52.7%) considered a psychiatric euthanasia procedure to be compatible with a psychiatric care relationship. In addition, 58.1% agreed that potentially effective therapeutic treatment options in the future should be taken into account during the euthanasia assessment and 80.9% supported the idea that the assessment should focus on the APC’s whole life, and not only on their medical state. As regards suicide, 43.7% of the psychiatrists agreed that euthanasia is an acceptable alternative to prevent the APC from attempting suicide.

Opinions were divided over whether or not physician-assisted suicide is more acceptable than euthanasia. Finally, over two thirds of psychiatrists (68.9%) agreed that in some cases the APCs’ euthanasia request was not assessed as thoroughly as they could be.

The open question at the end of the survey allowed psychiatrists to elaborate on and clarify their answers. Opposite motives (e.g. a psychiatrist’s own norms and values) as regards whether or not euthanasia in APC should remain legal were reported. But irrespective of normative disagreement on that statement, the answers to the open questions revealed scepticism and negative experiences regarding current euthanasia practice concerning APC in terms of a perceived insufficiency of due diligence and care by some colleagues during the assessment procedures.

As listed in Table 3, the results revealed no significant associations between a psychiatrist’s attitude to euthanasia remaining legal in APC and their personal or professional characteristics in terms of sex, perceived competence, work setting and work experience.

**Table 3:** Psychiatrists’ attitude towards euthanasia for psychiatric patients related to their personal and professional characteristics

	N	Mean	SD	F	p-value	95 CI	
						Lower Bound	Upper Bound
**Sex**							
Male	93	3.674	.215	.424	.516	3.249	4.100
Female	74	3.804	.173			3.462	4.146
**Perceived Competence**							
No	86	3.657	.205	.003	.959	3.251	4.063
Yes	81	3.825	.191			3.449	4.202
**Work Setting**							
Community-based	52	3.451	.274	2.832	.095	2.908	3.993
Hospital-based	115	3.963	.127			3.713	4.214
**Work Experience**							
< 5–10 years (group 1)	51	3.704	.297	.397	.673	3.117	4.291
11–20 years (group 2)	39	4.026	.273			3.486	4.566
> 20 (group 3)	77	3.510	1.44			3.225	3.795

^a^ Dependent Variable: Euthanasia should remain legally allowed for psychiatric patients

^b^ R Squared = .181 (Adjusted R Squared = .076)

### Readiness to engage actively in euthanasia procedures concerning APC

All psychiatrists, including those who have never been confronted with an explicit euthanasia request from APC in their professional career, were asked whether they could imagine being actively involved in such procedures in the future and if so, what type of role they would assign to themselves. From the 184 psychiatrists who answered the statements on euthanasia, 178 also answered these questions. As illustrated in Table 4, twenty-nine ﻿ (16.3%) were not willing to be involved in any active role during a euthanasia procedure concerning an APC in the future. Among those who would consider being involved in such euthanasia procedures (83.7%), respectively 68 and 43.8% would consider for themselves the role of referring or preliminary advising physician (See Box 2 in OSF idem for the English version of the glossary); 30.3% would consider being involved as the legally required first or second advising physician. A minority (8.4%) would engage in the performance of euthanasia with their own patient (8.4%) or a colleague physician’s patient (4.5%).

**Table 4:** Psychiatrists’ Readiness to be involved in the assessment of Psychiatric Euthanasia procedures

Readiness^b^	*N* = 178^a^
Would you consider to actively engage in one or more roles concerning explicitly expressed (distinct?) euthanasia requests of adult patients with (a) psychiatric disorder(s)?	N (%)
No, in not one single role	29 (16.3%)
Yes, as treating physician, who refers the own patient to a colleague-physician for further clarification/advise	121 (68.0%)
Yes, as attending physician, engaged in the clarification of a euthanasia request of my own patient	70 (39.3%)
Yes, as attending physician, engaged in the clarification of a euthanasia request of a colleague-physician’s patient	62 (34.8%)
Yes, as preliminary advising physician concerning a partial aspect (e.g. ruling out the existence of an acute depression, assessing mental competence).^c^	78 (43.8%)
Yes, as procedural advising physician concerning the legally required 1st or 2nd advice	54 (30.3%)
Yes, as performing physician, when being present at, assisting in of carrying out the act of euthanasia in my own patient	15 (8.4%)
Yes, as performing physician, when being present at, assisting in of carrying out the act of euthanasia in a colleague’s patient	8 (4.5%)

^a^ Missing cases *n* = 6: these missings concern psychiatrists who have filled out the online survey up to and including the 13 statements, but no(t much) further. It concerns psychiatrists that worked as psychiatrist with adult patients during the last 12 months (no retired or child psychiatrists)

^b^ More than one conceivable role could be ticked by the psychiatrists

^c^ In some cases, the ‘Advising role in a preliminary stage’ was chosen by retired psychiatrists and/or members of ethical committees

Table 5 represents the relation of a psychiatrist’s characteristics to their readiness to engage in euthanasia procedures concerning APC. Male and female psychiatrists did not differ significantly in their readiness to be involved in the assessment and/or performance of euthanasia with APC. However, those who felt more competent to be involved in euthanasia procedures more often indicated that they were prepared to consider an active role (*χ*^*2*^_*(1175)*_ = 5.140, *p* = .023), to give preliminary advice or legally required formal advice, (*χ*^*2*^_(*1,175)*_ = 10.654, *p* = .001 and *χ*^*2*^_(*1,175)*_ = 26.771, *p* = .000, respectively), and to consider a role as an attending physician (*χ*^*2*^_(*1175)*_ = 9.498, *p* = .002) in an APC’s euthanasia procedure.

**Table 5:** Psychiatrists’ readiness to be engaged in psychiatric euthanasia assessment related to their personal and professional characteristics

	Can conceive of themselves in the role of … *					
	NO ROLE	Referring physician	Preliminary advising physician	Formal advising physician	Attending physician	Performing physician
Sex	*p* = .060/.067	*p* = .084/.102	*p* = .873/.878	*p* = .098/.134	*p* = .543/.645	*p* = .849/1.000
Male (*n* = 98)	21 (21.4%)	61 (62.2%)	43 (43.9%)	35 (35.7%)	49 (50.0%)	10 (10.2%)
Female (*n* = 75)	8 (10.7%)	56 (74.7%)	32 (42.7%)	18 (24.0%)	41 (54.7%)	7 (9.3%)
Age	***p =*** **.000**	***p =*** **.000**	*p* = .091	*p* = .648	*p* = .091	*p* = .106
< 40 (*n* = 65)	**4 (6.2%)**	**60 (92.3%)**	35 (53.8%)	17 (26.2%)	38 (58.5%)	9 (13.8%)
41–60 (*n* = 64)	**8 (12.5%)**	**42 (65.6%)**	24 (37.5%)	20 (31.3%)	35 (54.7%)	7 (10.9%)
> 60 (*n* = 47)	**17 (36.2%)**	**17 (36.2%)**	17 (36.2%)	16 (34.0%)	18 (38.3%)	1 (2.1%)
Years experience	***p*** **= .004**	***p =*** **.000**	***p*** **= .003**	*p* = .240	*p* = .142	***p*** **= .022**
< 10 (*n* = 51)	**3 (5.9%)**	**47 (92.2%)**	**32 (62.7%)**	17 (33.3%)	32 (62.7%)	**9 (17.6%)**
10–20 (*n* = 41)	**4 (9.8%)**	**30 (73.2%)**	**13 (31.7%)**	8 (19.5%)	21 (51.2%)	**5 (12.2%)**
> 20 (*n* = 84)	**22 (26.2%)**	**42 (50.0%)**	**31 (36.9%)**	28 (33.3%)	38 (45.2%)	**3 (3.6%)**
Perceived Competence	***p*** **= .023/.026**	*p* = .282/.334	***p*** **= .001**	***p = .*000**	***p*** **= .002**	*p* = .211/.307
Yes (*n* = 88)	**9 (10.2%)**	56 (63.6%)	**49 (55.7%)**	**42 (47.7%)**	**56 (63.6%)**	11 (12.5%)
No (*n* = 87)	**20 (23.0%)**	62 (71.3%)	**27 (31.0%)**	**11 (12.6%)**	**35 (40.2%)**	6 (6.9%)

^a^ More than one conceivable role could be ticked by the psychiatrists

Note: In bold: significant p-values for Chi-Square tests/Fisher Exact tests

In Grey: One or more cells with expected count less than 5 and thus Chi^2^ does not have sufficient power

Years of work experience and older age were significantly and positively associated with not considering an active role (*χ*^*2*^_(*2,176)*_ = 11.239, *p* = .004 for work experience and *χ*^*2*^_(*2,176)*_ = 18.614, *p* = .000 for age range), whereas fewer years of work experience and a younger age were significantly and positively associated with referring the psychiatric patient to a colleague for the clarification of the euthanasia request (respectively *χ*^*2*^_(*2,176)*_ = 38.765 and *χ*^*2*^_(*2,176)*_ = 26.456, *p* = .000). Different ranges in years of work experiences were also statistically significant in considering an active role as preliminary advising physician (*χ*^*2*^_(*2176)*_ = 11.908, *p* = .003).

Anecdotal evidence from the answers to the open question at the end of the survey revealed that the readiness to be actively engaged in a euthanasia procedure concerning APC was based on the following motives: 1) moral and/or religious objection or agreement; 2) concerns regarding the difficulties of these euthanasia decision-making procedures, and 3) concerns about irreconcilable differences with and/or the inappropriate approaches of a colleague physician in current euthanasia practice concerning APC. Reported difficulties in the euthanasia decision-making procedures concerned: 1) some of the legal criteria being vague, as well as doubts about reconciling some legal criteria, such as a medically hopeless situation, due to subjectivities inherent in psychiatry, 2) the influence of transference and countertransference; 3) the lack of adequate courses and training on end-of-life education in regular and post-academic education and 4) concerns about how to reconcile the assessment of an APC’s euthanasia request with the current treatment of their psychopathology.

## Discussion

### Summary of results

This is the first survey in Belgium to study specifically the attitudes and readiness of Dutch-speaking psychiatrists regarding their involvement in euthanasia requests from APC. Almost three-quarters of Dutch-speaking psychiatrists supported the option of euthanasia as a legal end-of-life choice for APC. However, only half would consider an euthanasia assessment to be compatible with a therapeutic relationship and approximately one third (especially the younger generation) would engage in the concrete assessment of euthanasia cases concerning APC. Where active engagement was considered, an informal referring or preliminary advising role in the background was preferred to a formal role as the legally required advising physician, let alone as the performing physician. Finally, concerns were expressed regarding today’s euthanasia practice in terms of due diligence and care in the assessment of an APC’s euthanasia request.

### Strengths and limitations

As outlined above, we carefully constructed and pre-tested a survey, building on existing questionnaires and involving experts from the academic and the clinical psychiatric field, and tested for cognitive validity with a small group of psychiatrists. This pre-test phase resulted in feedback in both form and content. However, we cannot exclude the possibility of misunderstandings remaining as regards the interpretation of individual items.

In order to maximise response rates, we approached our psychiatrists by means of multiple response-inducing techniques [25]. A fair response rate of 40% was achieved (considering the target group of psychiatrists and the delicacy of the topic) [26] and rich quantitative as well as qualitative data were obtained.

However, the results from this study with a 40% response rate from a sample of VVP-affiliated psychiatrists cannot readily be generalized to the full population of psychiatrists in the Dutch-speaking region of Belgium, and must therefore be interpreted with caution. There are avid supporters of as well as opponents of euthanasia for psychiatric patients, and the societal debate on euthanasia itself extends to psychiatrists as part of society. Keeping in mind the extremely sensitive nature of our research topic, we could have missed the answers of psychiatrists positioned at either end of the euthanasia debate, i.e. the ones strongly opposed to the study and its set-up as well as the ones strongly opposing critical reflections on today’s euthanasia practice. However, our study findings revealed both support for maintaining the current law as well as the identification of various scopes of improvement that - in the long run – could lead to sufficiently built-in safeguards integrated to protect against potential wrongdoings. By doing so, our study may contribute to a proper debate about the most appropriate euthanasia practices and as a consequence, may be seen as the first step in order to restore the current lack of trust in and negative experiences of some colleague-physicians in this field.

That said, there is reason to believe that we have minimized the risk of a biased sample. According to email correspondence with the VVP, their database membership comprises an estimated 80 to 90% of all Dutch-speaking psychiatrists. If we extrapolated our sample of 184 Dutch-speaking psychiatrists working with APC to the estimated sample of all Dutch-speaking psychiatrists working with APC (around 600 in total), it would mean that we had reached close to one third (184/600) of all Dutch-speaking psychiatrists working with APC.

However, it should be noted that the majority of responding psychiatrists were professionally active in a hospital-based setting rather than in a community-based setting. Taking into account that this does not necessarily reflect the general division of private versus hospital-based practices in Flanders (nor in other countries), this suggests that the topic of euthanasia in APC is more pervasive in hospital-based practices, likely due to more severe (consequences of) psychopathology as well as the requirement to engage in intensive treatment programs before APCs can be considered to be in a medically hopeless situation and eligible for euthanasia. This should be further examined in future research.

### Interpretation of findings

Contrary to Canadian and Swiss findings [17, 18] but in line with Dutch findings [15], the majority of psychiatrists were in favour of continuing to allow APC to die by means of euthanasia. Most Dutch-speaking psychiatrists agreed that APC can effectively meet the substantive legal criteria, although this mainly concerns the criteria that can be attributed directly to the patient (e.g. mental competence, unbearable suffering), and to a somewhat lesser degree the criteria attributed to the medical condition (e.g. medically hopeless situation and reasonable treatment perspectives). However, almost one out of three psychiatrists was opposed to euthanasia for psychiatric reasons. This could be due partially to fundamental ethical and religious objections, insufficient competence in handling such requests at a *practical clinical and ethical* level or a desire for additional or more stringent legal criteria for this specific patient group. As for the latter, the guidelines for adequate euthanasia assessment in APC have only recently been published, so their impact is still unknown (i.e. whether or not these guidelines were sufficiently known and/or sufficiently address the difficulties in the euthanasia decision-making procedure).

Although the majority of psychiatrists were in favour of euthanasia remaining legal for this patient group, only a minority were willing to be actively engaged in it due to the difficulty of the decision-making procedure, e.g. the vagueness of the law and the subjectivities inherent in the medical discipline of psychiatry. However, compared to the Dutch findings, the percentage of psychiatrists supporting this legal option for APC and willing to engage in such procedures was slightly higher in Flanders than in the Netherlands (74.5% versus 70.5 and 84% versus 82% respectively), which may be due to the inclusion of trainees in psychiatry in the sample or to differences in the respective legal end-of-life frameworks. For example, the Belgian law provides stricter legal criteria as regards the non-terminally ill, which may provide more guidance to rely on. It also explicitly assigns a specific role to psychiatrists, as the consultation of at least one psychiatrist is required for euthanasia assessment purposes in APC [27]. In addition, most psychiatrists would rather refer APC to a colleague for the clarification of a euthanasia request where it was deemed difficult to reconcile with the treatment of their psychopathology or with their rehabilitation. On the other hand, conscientious objection by the psychiatrist is also legally accepted and does happen, as shown in our study. This raises questions about how patient referral is organized. Given that only a minority of psychiatrists are willing to engage actively in such euthanasia procedures, it is important to ensure that APC are able to have their euthanasia request heard. The consequences for APC can be increased suffering and potential suicide (or suicide attempts) when the APC are unable to discuss their wishes concerning death.

It is striking that almost three quarters of the psychiatrists had questions about the approaches of certain colleagues to euthanasia practice in APC. Some expressed concerns about what they saw as the overly permissive approaches taken at end-of-life consultation centres. As only a minority of psychiatrists were willing to engage in the assessment of an explicit euthanasia request from APC, and mostly only as the referring physician, their patients may find their way to these other psychiatrists and centres. However, the concerns expressed about euthanasia requests that are dealt with too lightly may also be interpreted the other way around, as the answers to the open questions also showed worries about some psychiatrists dismissing euthanasia requests too quickly.

### Implications for practice, policy and research

More research is needed to further examine the underlying motives influencing the attitudes of psychiatrists towards, and readiness to deal with, euthanasia requests from APC, and to gain insight into the reasons for the discrepancy between their attitudes towards and their readiness to be involved in these cases. Is the discrepancy primarily due to a need for more qualitative education and/or do the legal requirements need more clarification? Further research might reveal more potential study associations between psychiatrists’ profiles (e.g. psychiatrists’ values in life, beliefs, religiosity and norm systems) in the context of their attitudes and/or readiness to engage in euthanasia assessment regarding APC. In addition, in-depth qualitative studies could further expand our insights into psychiatrists’ concrete experiences with such euthanasia requests and assessment procedures.

What can be learned from the lack of trust in other physicians – on both sides of the spectrum – and negative experiences with them, in order to find adequate ways to establish and/or restore this much-needed trust? Our results suggest that psychiatrists who feel sufficiently competent in the assessment of euthanasia requests are more likely to be actively engaged in them as the preliminary or formally advising physician or the attending physician. In this respect, it is noteworthy that only a minority of psychiatrists have received specific training in medical end-of-life decisions, which could affect their attitudes towards euthanasia in these patients, their perception of their own capacity to engage in them and their lack of trust in and collaboration with experienced colleagues and end-of-life centres. The reasons why the younger generation of psychiatrists seemed more prepared to engage actively in these procedures should also be addressed, as they may give insight into and influence future euthanasia practice concerning APC.

Future cross-national research could provide important insights into the determinants of legal and medical culture regarding differences in end-of-life decisions in different countries, and how they affect the current medical practice of euthanasia. Recommendations for policy and practice arising from this study include budgeting for more in-depth evaluation studies (e.g. to gain insight into the barriers that impede psychiatrists from engaging) and other support that could increase the quality and transparency of today’s euthanasia practice with APC, increasing all actors’ levels of confidence in this practice, whether by education or by further clarification of the legal requirements.

## Conclusions

Although the majority of Flemish psychiatrists indicate that euthanasia should remain legally permissible for APC where the current legal criteria are met, a minority (one third) is prepared to be actively engaged in the assessment of a euthanasia request and fewer than 10% are willing to assist in the administration of the lethal drugs to the APC.

## Data Availability

This study is fully disclosed, except for the database for reasons of anonymity and privacy. To access the supplementary materials, see the Open Science Framework repository at (https://osf.io/fg3ys/). Upon publication of this paper, the repository will be made public and a shorter link will be provided.

## Abbreviations

APC: Adults with Psychiatric Conditions
